# Supplementary material for: Association between oral microbiome and five types of respiratory infections: a two-sample Mendelian randomization study in east Asian population
Source: Front Microbiol. 2024 Apr 10;15:1392473. doi: 10.3389/fmicb.2024.1392473 (PMC11039966; doi:10.3389/fmicb.2024.1392473)
Supplement: Supplementary file 1 [file Data_Sheet_1.zip › Supplementary Materials/MR plots of saliva/Chronic sinusitis/s__Lancefieldella_sp000564995_mgs_2677/leave_one_out.pdf]

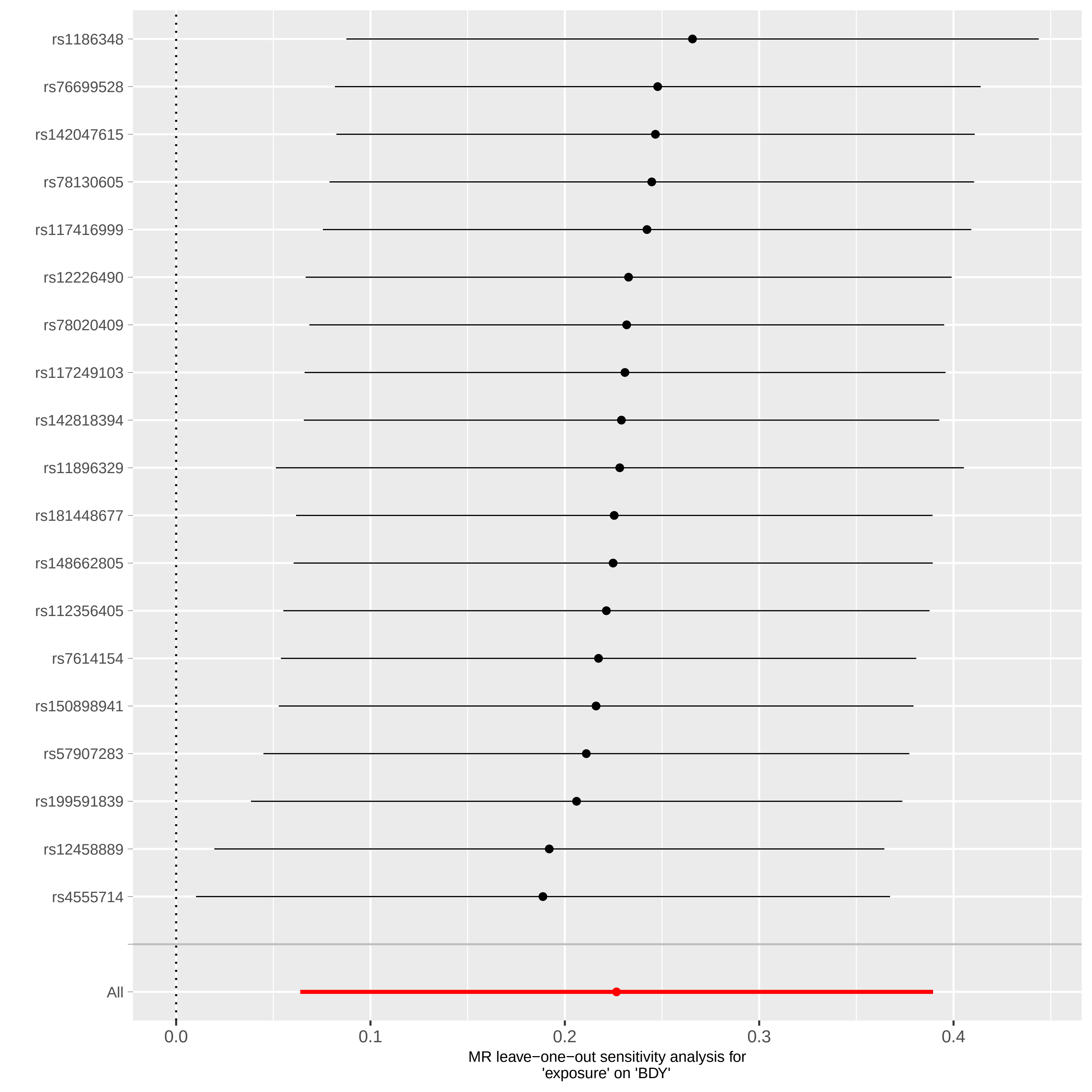
rs1186348
rs76699528
rs142047615
rs78130605
rs117416999
rs12226490
rs78020409
rs117249103
rs142818394
rs11896329
rs181448677
rs148662805
rs112356405
rs7614154
rs150898941
rs57907283
rs199591839
rs12458889
rs4555714
All
0.0
0.1
0.2
0.3
0.4
MR leave−one−out sensitivity analysis for
'exposure' on 'BDY'
